# The deposition and wet etching of Mg-doped ZnO films and their applications for solidly mounted resonators

**DOI:** 10.1039/d0ra00659a

**Published:** 2020-03-06

**Authors:** Chengzhang Han, Haoran Ma, Yanping Wang, Jing Liu, Lihua Teng, Hao Lv, Qiuling Zhao, Xia Wang

**Affiliations:** Optoelectronic Materials and Technologies Engineering Laboratory, Shandong, Physics Department, Qingdao University of Science and Technology Qingdao 266042 China sdqlzhao@163.com phwangxia@163.com; College of Mechanical and Electronic Engineering, Qingdao Binhai University Qingdao 266555 China

## Abstract

In this report, a solidly mounted resonator (SMR), consisting of an Au electrode, Mg-doped ZnO (Mg_*X*_Zn_1−*X*_O) piezoelectric film and Bragg acoustic reflector, was fabricated on a Si substrate by radio frequency (RF) magnetron sputtering. As a key processing step for the SMR, Mg_*X*_Zn_1−*X*_O films with high *c*-axis orientation were fabricated and the crystalline structure, surface morphology and roughness of the films were investigated. The surface morphology, optical transmittance and shape control of Mg_*X*_Zn_1−*X*_O films were investigated by the chemical wet-etching method with various etchants. The profiles and line patterns of Mg_*X*_Zn_1−*X*_O films etched with FeCl_3_·6H_2_O solutions are satisfactory and fully meet the industrial requirements. The Bragg acoustic reflector, made entirely of metal, has small internal stress and good heat conduction. An SMR based on a Mg_*X*_Zn_1−*X*_O piezoelectric film shows a resonant frequency of 2.402 GHz, and the *k*_eff_^2^, *Q*_S_ and *Q*_P_ of the SMR are 3.07%, 415 and 546, respectively.

## Abstract 2

In this report, a solidly mounted resonator (SMR), consisting of an Au electrode, Mg-doped ZnO (Mg_*X*_Zn_1−*X*_O) piezoelectric film and Bragg acoustic reflector, was fabricated on a Si substrate by radio frequency (RF) magnetron sputtering.

## Graphical abstract

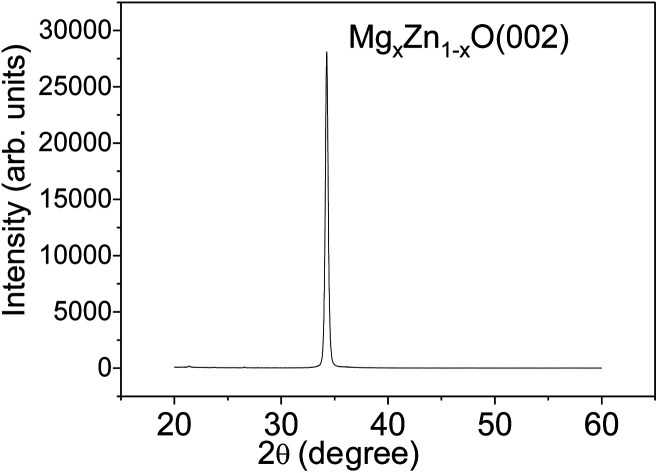

## 1. Introduction

With the advancement in micro/nano fabrication, film bulk acoustic resonators (FBARs) have been proposed as typical micro-electromechanical system (MEMS) piezoelectrical devices.^1–4^ As bulk acoustic wave (BAW) devices operating in the GHz range, FBARs have attracted much attention due to their small size, high operating frequency and potential applications in high-frequency communication and mass-sensitive sensor areas.^5–8^ As a kind of FBAR, solidly mounted resonators (SMRs) are composed of a piezoelectric layer sandwiched between electrodes and Bragg reflector consisting of alternating high and low acoustic impedance quarter-wavelength thick dielectric or metallic layers.^9,10^ The SMR, with good mechanical strength and excellent acoustic properties, and being closer to CMOS integration, was therefore chosen in this work.^11,12^

In recent decades, owing to excellent piezoelectric property, better quality factor, and high electromechanical coupling coefficient, ZnO is becoming a very promising candidate for FBAR devices as a piezoelectric material.^13–16^ However, ZnO have the drawback of low longitudinal acoustic wave velocity and low resistance, which limits its application to high sensitivity acoustic sensors.^17^ Mg_*X*_Zn_1−*X*_O, a ternary compound formed by alloying ZnO and MgO, has attracted more and more attention due to its special properties such as higher acoustic velocity and resistance than that of ZnO.^18–20^ Studies have shown Mg^2+^ is doped into the ZnO crystal by substituting the Zn^2+^ position and Mg_*X*_Zn_1−*X*_O still maintains the wurtzite crystal structure when the percentage of Mg atoms is less than 33%.^21^ By controlling the percentage of Mg-doped in the material, the Mg_*X*_Zn_1−*X*_O films with satisfactory sound velocity and electromechanical coupling coefficient can be tailored.^22^

Mg_*X*_Zn_1−*X*_O films have been fabricated by a variety of techniques such as magnetron sputtering,^23,24^ atomic layer deposition,^25,26^ spray pyrolysis,^27,28^ pulsed laser deposition (PLD),^29,30^ and sol–gel coating.^31,32^ Among these deposition techniques above, magnetron sputtering might be a most practical method because of its high deposition rate, good adhesion and good uniformity, which is suitable for commercialization.^33,34^ In generally, ZnO thin films can be easily and fast etched by various common etchants such as HCl, HNO_3_, H_2_SO_4_, H_3_PO_4_ and so on.^35,36^ However, anisotropic etching profiles could be generally obtained and the surface shapes are too difficult to control in the acid solutions through a number of experiments, which has a negative impact on etching patterning. Recently, ferric chloride (FeCl_3_·6H_2_O) as a kind of representative etchants, has been carried out to solve this problem.^37,38^

In this paper, we fabricated Mg_*X*_Zn_1−*X*_O films with high *c*-axis orientation by RF magnetron sputtering and characterized structure and surface morphology of Mg_*X*_Zn_1−*X*_O films. In addition, we explored the effects of FeCl_3_·6H_2_O as a novel etchant on surface morphology, optical transmittance and shape control of Mg_*X*_Zn_1−*X*_O films. The novel Bragg acoustic reflector, made entirely of metal, has small internal stress and good heat conduction. We fabricated a SMR based on Mg_*X*_Zn_1−*X*_O films and Bragg acoustic reflector with the optimized fabrication condition and investigated the performance of SMR.

## 2. Experiment

### 2.1 Mg_*X*_Zn_1−*X*_O films deposition

The Mg_*X*_Zn_1−*X*_O thin films were deposited on (100) oriented silicon (Si) substrates in the RF magnetron sputtering system with a base pressure of 5 × 10^−5^ Pa. Mg_*X*_Zn_1−*X*_O (*X* = 10%) was used as a target source material and a mixture of argon (Ar) (99.999%) and oxygen (O_2_) (99.999%) was used as sputtering gas. The distance target-to-substrate is about 70 mm and the target diameter is 80 mm.

### 2.2 Mg_*X*_Zn_1−*X*_O films wet etching

The sputtered Mg_*X*_Zn_1−*X*_O films were standard photolithography processed by a designed pattern mask. About 1 μm thickness positive photoresist (AZ 4620) was coated on the surface of the Mg_*X*_Zn_1−*X*_O films by three-step spin coating. After prebaked at 100 °C for 1 min, a mask aligner was used to transfer the pattern design on the mask to the photoresist. After that, the Mg_*X*_Zn_1−*X*_O films with patterned photoresist was dipped in the developer solution (AZ 400K) to remove the exposed photoresist, washed and post-baked at 100 °C for 5 min.

To acquire patterned Mg_*X*_Zn_1−*X*_O films, the wet etching was carried out in dilute HCl, C_2_H_4_O_2_ and FeCl_3_·6H_2_O aqueous solution prepared at the concentration values of 0.001 mol l^−1^, 0.01 mol l^−1^, and 0.1 mol l^−1^, respectively. All wet etchings were carried out in an ambient temperature kept at 25 °C and etching time was precisely controlled. After the wet etching process, Mg_*X*_Zn_1−*X*_O films were immediately washed by deionized water and dried by nitrogen.

### 2.3 SMR fabrication

The procedures of the SMR fabrication were be divided into three steps: Bragg reflector deposition, Mg_*X*_Zn_1−*X*_O films fabrication, and top electrode deposition. The Bragg reflector, consisting of Ti and W layers, was first deposited on p-type 3 inch Si (100) substrate with 1–10 Ω cm resistivity at 25 °C, and then Mg_*X*_Zn_1−*X*_O films were deposited on the top of the Bragg reflector at 300 °C. Lastly, a Au film was deposited on top of Mg_*X*_Zn_1−*X*_O films for top electrode. Similar to Mg_*X*_Zn_1−*X*_O films, Ti, W, and Au film were also obtained by the RF magnetron sputtering system using Ti, W, and Au targets in a pure Ar atmosphere, respectively. The distance target-to-substrate is about 70 mm and the diameters of all targets are 80 mm. Finally, a thermal annealing process at 300 °C was also performed to relieve the stress in multilayer films to improve the performance of the SMR.

### 2.4 Characterizations

The crystalline structure of Mg_*X*_Zn_1−*X*_O films was investigated by X-ray diffraction (XRD, Bruker Advanced D8) using a Cu-Kα radiation (*λ* = 1.54187 Å) in a *θ*–2*θ* scanning mode. The surface morphology and cross-sectional morphology of Mg_*X*_Zn_1−*X*_O films were observed by a field emission-scanning electron microscope (FE-SEM, Carl Zeiss Ultra55). The surface roughness (RMS) of Mg_*X*_Zn_1−*X*_O films was investigated by atomic force microscopy (AFM, Nanofirst 3000). The optical transmission of Mg_*X*_Zn_1−*X*_O films was measured by UV spectrophotometer (PerkinElmer Lambda 750). The cross-sectional morphology of SMR was observed using a FE-SEM. Finally, the frequency response of SMR was measured by *S*-scattering parameters with a probe station (Cascade EPS 150 RF) and a network analyzer (HP 8712E). All measurements of SMR were carried out in an ambient temperature kept at 25 °C.

## 3. Results and discussions

### 3.1 Mg_*X*_Zn_1−*X*_O films characterization

**Fig. 1:**
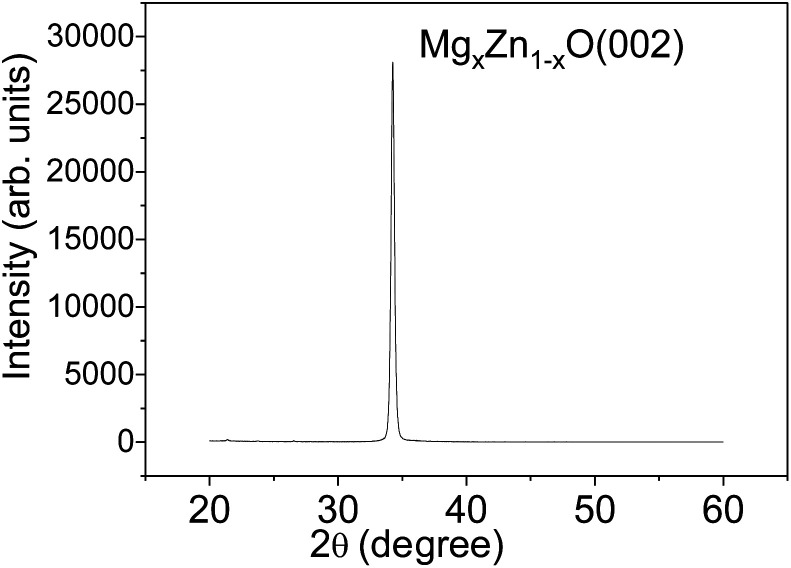
The XRD patterns of Mg_*X*_Zn_1−*X*_O films grown on silicon substrates.

The sputtering parameters have an important effect on the crystalline structure and crystalline grain size of Mg_*X*_Zn_1−*X*_O films, which determine the quality of films. After many experiments and measurements, the optimal sputtering parameters of Mg_*X*_Zn_1−*X*_O films with high *c*-axis orientation and all metal materials in experiments were summarized in Table 1. Fig. 1 shows the XRD patterns of Mg_*X*_Zn_1−*X*_O films grown on silicon substrates. For the Mg_*X*_Zn_1−*X*_O films, a very strong diffraction peak was observed at 34.4° with a full width at half-maximum (FWHM) of 0.30°, which corresponds to the diffraction from the Mg_*X*_Zn_1−*X*_O (002) plane. This indicates that the preferential Mg_*X*_Zn_1−*X*_O growth orientation is along the wurtzite *c*-axis and perpendicular to the surface of substrate.

**Table 1:** The specific sputtering parameters of Ti, W, Mg_*X*_Zn_1−*X*_O and Au

Sputtering parameters	Ti	W	Mg_*X*_Zn_1−*X*_O	Au
RF power (W)	150	150	250	150
Ar flow rate (sccm)	20	20	40	20
Sputtering pressure (Pa)	1.5	1.5	2.5	1.5
Substrate temperature (°C)	300	300	300	300

**Fig. 2:**
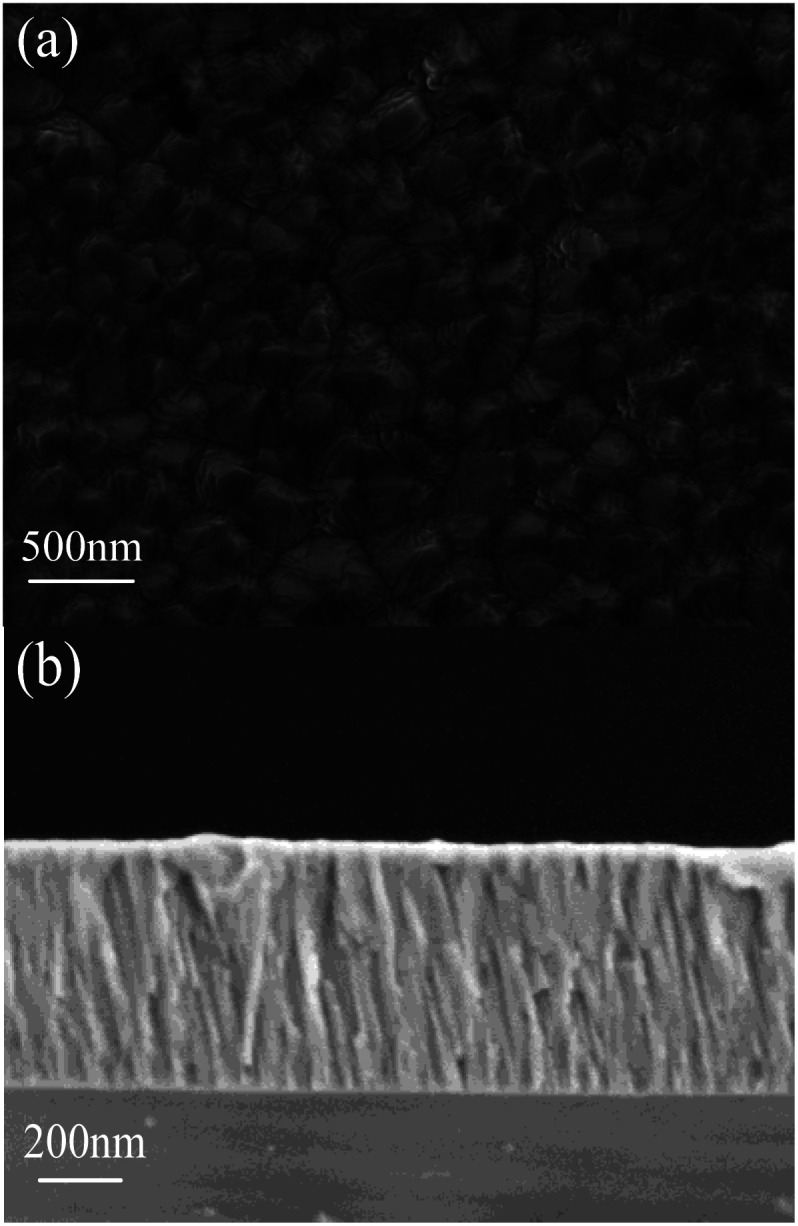
The SEM micrographs obtained for Mg_*X*_Zn_1−*X*_O films. (a) Surface image, (b) cross-sectional image.

Fig. 2 shows the SEM surface and cross-sectional images of Mg_*X*_Zn_1−*X*_O films deposited on silicon substrate. The Mg_*X*_Zn_1−*X*_O films demonstrate clearly a hummock-like surface morphology with smooth, homogeneous, uncracked, compact and dense. The surface morphology consists of a large number of hexagonal crystalline grains with no visible pores and defects over the films in Fig. 2(a). The Mg_*X*_Zn_1−*X*_O films perpendicular to the surface of silicon substrate exhibit highly oriented and compact columnar structure. Besides, the grain boundaries among columns in films and the good cohesion between the Mg_*X*_Zn_1−*X*_O films and silicon substrate are observed in Fig. 2(b), obviously.

**Fig. 3:**
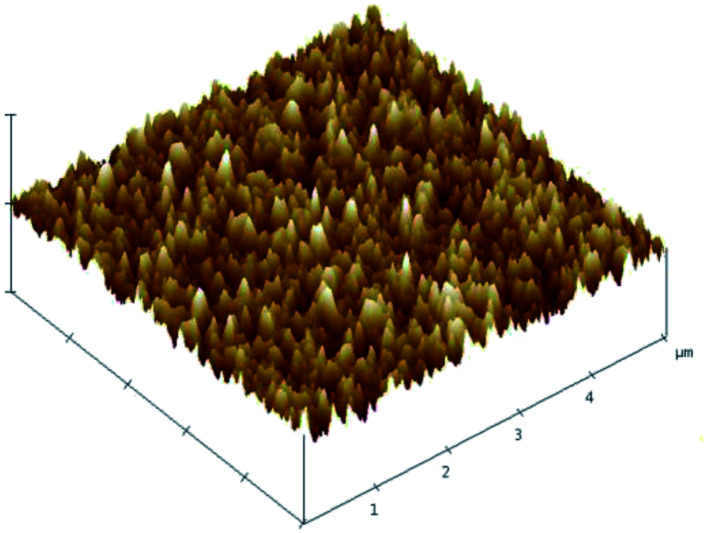
Three-dimensional AFM image of Mg_*X*_Zn_1−*X*_O. films deposited on a silicon substrate.

Fig. 3 shows a three-dimensional AFM image of the Mg_*X*_Zn_1−*X*_O films deposited on silicon substrate. The surface roughness of the Mg_*X*_Zn_1−*X*_O films is 3.37 nm. The rounded and homogeneous grain shape can be observed from the picture, which reveals the fact that Mg_*X*_Zn_1−*X*_O films with a homogeneous smooth surface over the whole wafer. Low surface roughness is connected with low acoustic loss in Mg_*X*_Zn_1−*X*_O films, which is suitable for SMR.

### 3.2 Mg_*X*_Zn_1−*X*_O films etching characteristics

**Fig. 4:**
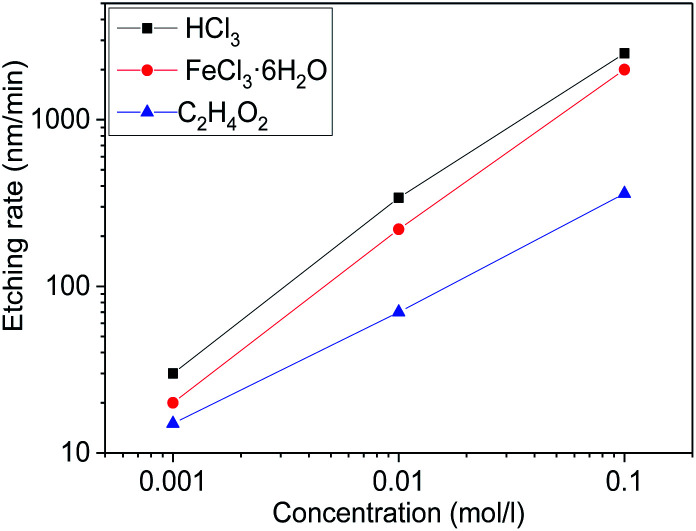
The functions of etching rates and concentrations of different etchant aqueous solutions.

The functions of etching rates and concentrations of different etchant aqueous solutions were shown in Fig. 4. It should be noticed that the etching rate of HCl is most efficient, the C_2_H_4_O_2_ is least, and the FeCl_3_·6H_2_O is in between. Under a wide range of etchant concentration, the etching rate almost linearly increases with etchant concentration. It is well known that the etching process of Mg_*X*_Zn_1−*X*_O in acid solution is the H^+^ ions attacking the oxygen atom site on the Mg_*X*_Zn_1−*X*_O structure, producing water and soluble salt.^39^ The higher concentration of H^+^ ions, the faster etching rate of Mg_*X*_Zn_1−*X*_O films. As a kind of strong electrolytes, HCl can be totally ionized to generate enough H^+^ ions and the concentration of H^+^ ions is the same as HCl in the aqueous solution, so the etching rate is the fastest. While in FeCl_3_·6H_2_O aqueous solution, Fe^3+^ can react with the H_2_O by hydrolysis and generate H^+^ ions. The hydrolysis of Fe^3+^ can be divided into three steps and H^+^ ions can be generated in every step, which provides much H^+^ ions to react with Mg_*X*_Zn_1−*X*_O films. Due to the hydrolysis is reversible, Fe^3+^ can not totally react with the H_2_O to generate H^+^ ions, so the concentration of H^+^ ions in FeCl_3_·6H_2_O solutions is lower than HCl solution. This is the reason why the etching rate of FeCl_3_·6H_2_O is lower than HCl. It is well known that the C_2_H_4_O_2_ is a kind of weak electrolytes, so only a part of C_2_H_4_O_2_ can be ionized to generate less H^+^ ions compared to HCl at the same mole concentration. As a result, the concentration of H^+^ ions is the lowest among the three solutions and the etching rate is lowest.

**Fig. 5:**
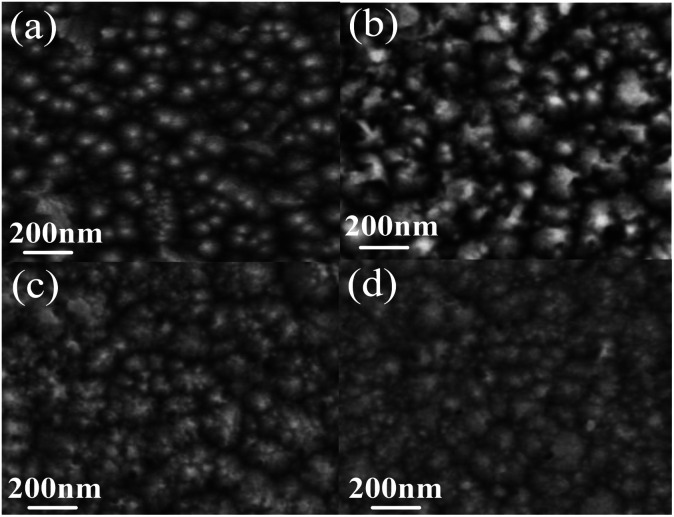
The SEM micrographs of surface texture Mg_*X*_Zn_1−*X*_O films etched in different etchants. (a) As-grown, (b) C_2_H_4_O_2_ solution, (c) FeCl_3_·6H_2_O solution, (d) HCl solution.

The SEM micrographs of surface texture Mg_*X*_Zn_1−*X*_O films etched in different etchants were illustrated in Fig. 5. The concentration of all etchants was controlled to be 0.01 mol l^−1^ and the etching time was controlled in 1 min. It is observed clearly that the etched samples show distinctive rough surface morphologies due to different etching rates of different etchants. The sample as-grown Mg_*X*_Zn_1−*X*_O films without etched consists of close-packed hummock-like crystals surface morphology with smooth, homogeneous and uncracked. By compared, hummock-like crystals are becoming gradually disappeared and the surfaces are becoming gradually rough and cracked with the increase of etching rate in different etchant solutions. It can be proved that surface texture of Mg_*X*_Zn_1−*X*_O films is the most severely damaged in HCl solution, the least damaged in C_2_H_4_O_2_ solution, and the moderate in FeCl_3_·6H_2_O solution.

**Fig. 6:**
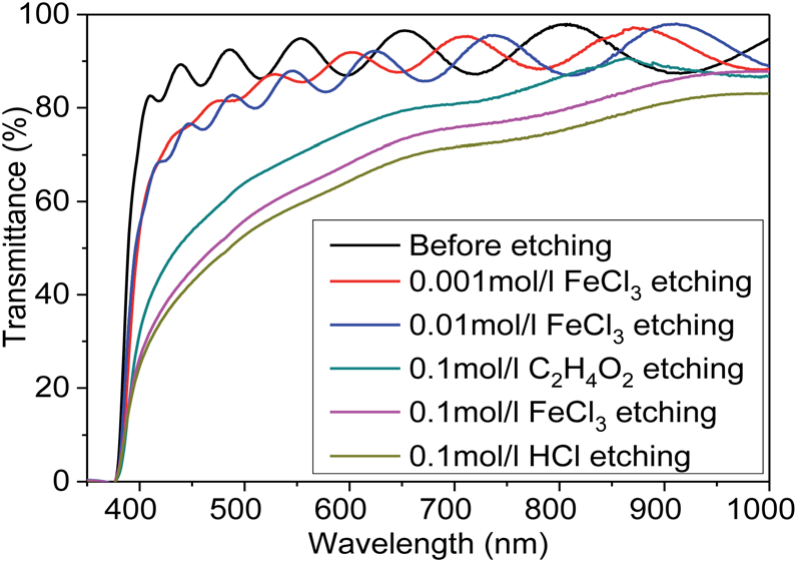
The optical transmittance of Mg_*X*_Zn_1−*X*_O films etched in different etchants.

Fig. 6 shows the optical transmittance of Mg_*X*_Zn_1−*X*_O films etched in different etchants. The thicknesses of Mg_*X*_Zn_1−*X*_O films deposited on glass were 1 μm and the etching time was controlled in 20 s. It was observed clearly that the optical transmittance decrease with the etching rates increase. The etchants destroy the surface morphologies of Mg_*X*_Zn_1−*X*_O films and lead to the changes of optical transmittance. The etching rates are faster, the morphologies of destruction are more serious, and the optical transmittances are lower. The results above show that the optical properties can be controlled by varying either etchants or etchants concentration.

**Fig. 7:**
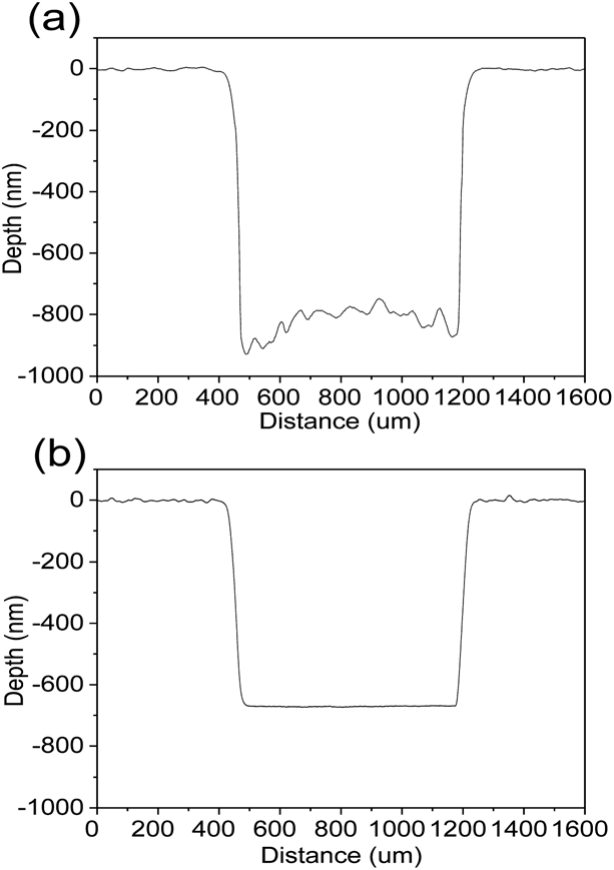
The comparison of etched profiles of the films. (a) Etched in HCl solutions. (b) Etched in FeCl_3_·6H_2_O solutions.

**Fig. 8:**
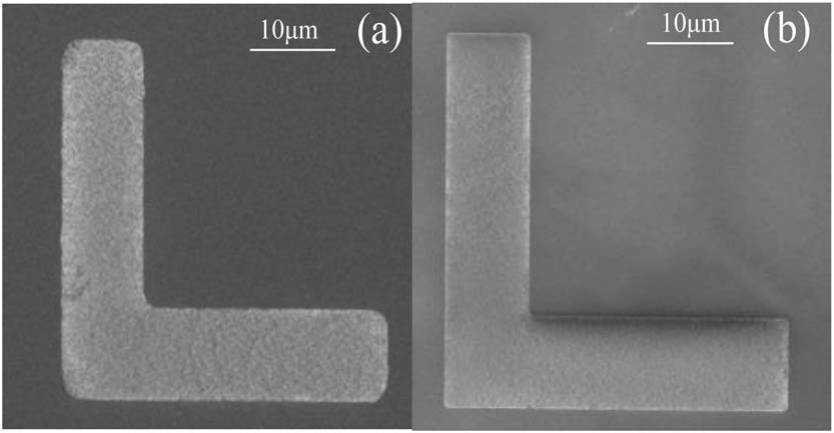
The comparison of right angle line pattern of the Mg_*X*_Zn_1−*X*_O films. (a) Etched in HCl solutions. (b) Etched in FeCl_3_·6H_2_O solutions.

Fig. 7 shows etched profiles of Mg_*X*_Zn_1−*X*_O films by different etchants at the same time. The concentration of etchants was controlled to be 0.01 mol l^−1^ and the etching time was controlled in 3 min. A rough and high in the middle and low on both sides etching profile is observed at the bottom of the step from Fig. 8(a), which results from the faster etching rate near the mask edge than that in the center in HCl solutions. Such kind of etching profile has an adverse effect on the shape and size of the devices and deteriorate devices performance. In contrast, a very smooth etching profile is observed clearly from Fig. 8(b), which results from the same etching rate near both the edge and center of the mask in FeCl_3_·6H_2_O solutions. The smooth etching profile in the etched area indicates that the etching rate is almost homogeneous over the entire sample.

In addition, Fig. 8 displays the etched right angle line patterns of Mg_*X*_Zn_1−*X*_O films in HCl and FeCl_3_·6H_2_O solutions. The right angle line patterns etched in HCl solutions displayed a smooth surface and sharp etching step without etching residue in the etched region in Fig. 8(a). But the right angles of the line patterns were destroyed seriously, which indicates that the etching rates along the different directions are not homogeneous on the sample surface. As shown in Fig. 8(b), the problem was settled satisfactorily by FeCl_3_·6H_2_O solutions. The right angles of the line patterns etched in FeCl_3_·6H_2_O solutions are pointed and integrated, which indicates the etching rates along the different directions are homogeneous on the sample surface and fully meets the requirement of shape and size of the devices.

### 3.3 SMR characterization

**Fig. 9:**
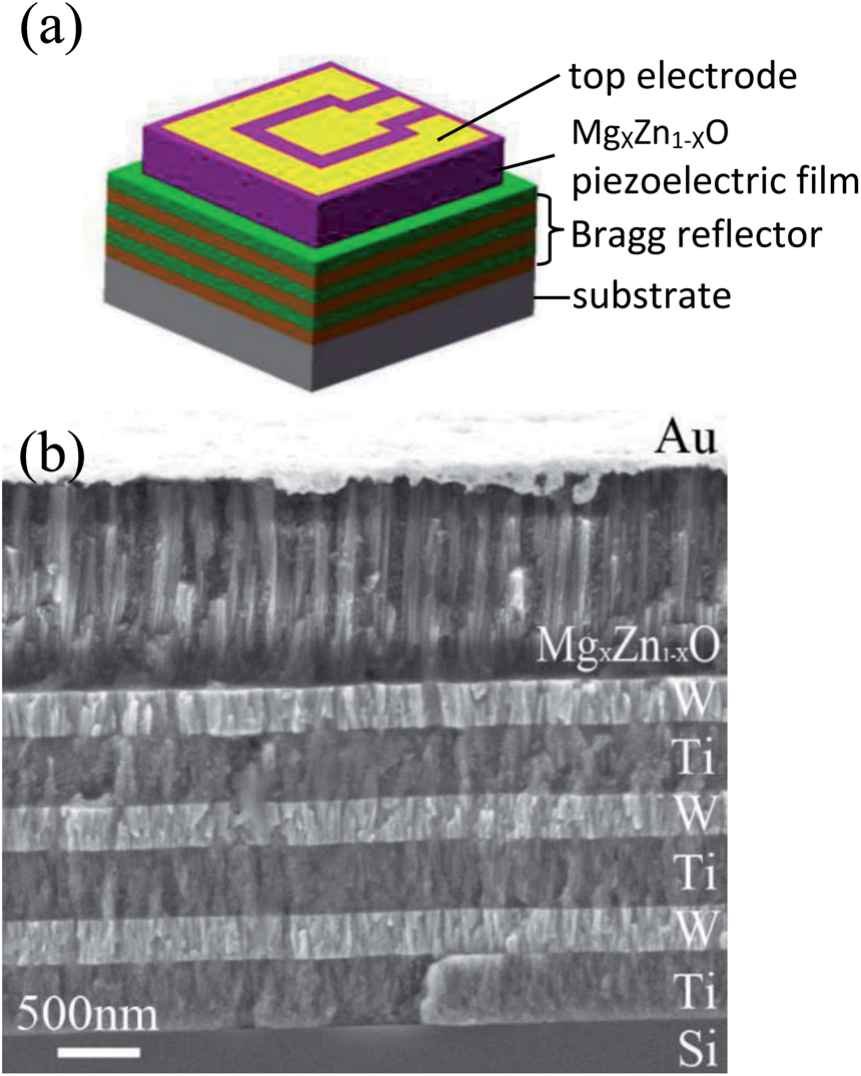
(a) The schematic illustration of SMR. (b) The cross-section view morphologies of integrated SMR.

Fig. 9(a) shows a 3D-view schematic illustration of SMR and Fig. 9(b) shows the cross-section view morphologies of integrated SMR with Au/Mg_*X*_Zn_1−*X*_O/Ti/W multilayer films structure. The Mg_*X*_Zn_1−*X*_O films perpendicular to Bragg reflector exhibit highly oriented and compact columnar structure. The interfaces between the Mg_*X*_Zn_1−*X*_O films and Bragg reflector are clearly visible and distinct, verifying that the different membrane layers are not diffusive with each other. As the Bragg reflector was made entirely of metal, it had small internal stress and good heat conduction. Note that top W layer of the Bragg reflector on the top of Si also served as the electrode for frequency measurements.

**Fig. 10:**
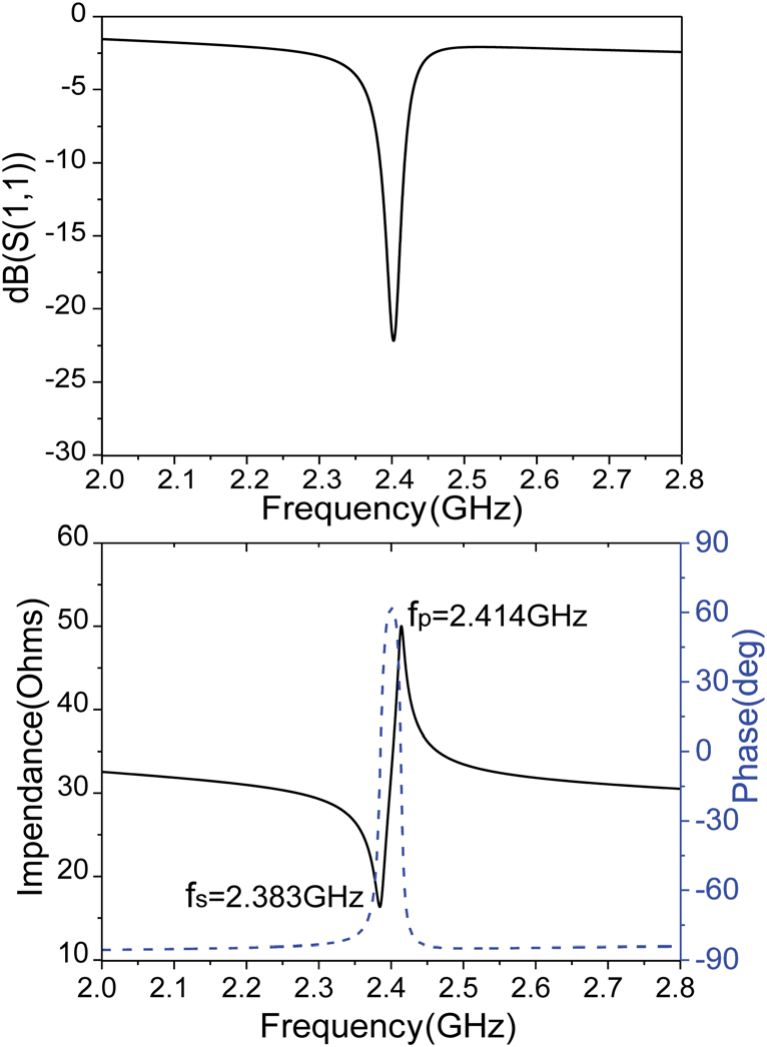
The reflection coefficient *S* (1,1), impedance and phase response of SMR.

The reflection coefficient *S* (1,1), impedance and phase response of SMR were measured with probe station and network analyzer and displayed in Fig. 10. The frequency response of SMR based on Mg_*X*_Zn_1−*X*_O films we fabricated is approximately around 2.402 GHz with a return loss of −24.57 dB, and the series resonant frequency (*f*_s_) and parallel resonant frequency (*f*_p_) appeared at 2.383 GHz and 2.414 GHz, respectively. Both the corresponding coupling coefficient *k*_eff_^2^ and quality factor *Q* values of SMR can be derived easily based upon the formula as follows:^40^1
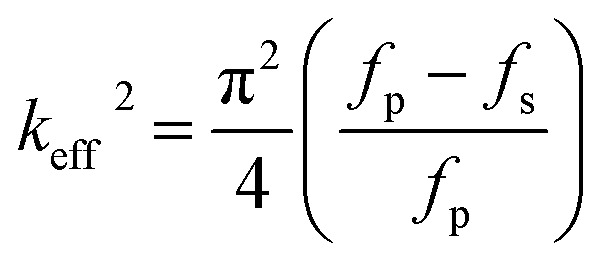
2
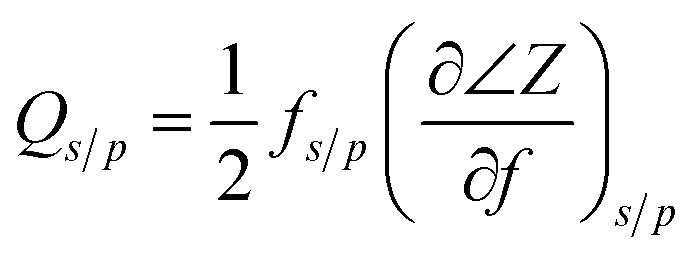
where *Q*_S_ and *Q*_P_ are parallel and series quality factors and *Z* is the input electrical impedance. According to calculation above, the *k*_eff_^2^, *Q*_S_ and *Q*_P_ of SMR we fabricated are 3.07%, 415, and 546, respectively.

## 4. Conclusions

In this paper, we fabricated Mg_*X*_Zn_1−*X*_O films with high *c*-axis orientation by RF magnetron sputtering and investigated the crystalline structure, surface morphology and roughness of films. The effects of different etchants on surface morphology, optical transmittance and shape control of Mg_*X*_Zn_1−*X*_O films were very obvious. The FeCl_3_·6H_2_O shows better etched profiles and right angle line patterns than other etchants and more suitable for the patterning Mg_*X*_Zn_1−*X*_O films. The SMR consisting of Mg_*X*_Zn_1−*X*_O films and Bragg acoustic reflector with a resonant frequency of 2.402 GHz, and the *k*_eff_^2^, *Q*_S_ and *Q*_P_ of SMR are 3.07%, 415, and 546, respectively. So SMR based on Mg_*X*_Zn_1−*X*_O piezoelectric films provides a new approach in the high-frequency communication and sensitive-mass sensor areas.

## Conflicts of interest

There are no conflicts to declare.

## Supplementary Material
